# Eating behavior, salt intake and health risks correlates in South Asian students

**DOI:** 10.1192/j.eurpsy.2026.11377

**Published:** 2026-07-31

**Authors:** O. V. Nikolaeva, E. N. Tashkenbaeva, J. N. Daminov, T. E. Nikolaeva

**Affiliations:** 1 Department of Hospital Therapy, I.N. Ulianov Chuvash State University, Cheboksary, Russian Federation; 2 Department of Internal Medicine and Cardiology No. 2; 3 Faculty of Pharmacy, Samarkand State Medical University, Samarkand, Uzbekistan; 4 Faculty of Medicine, I.N. Ulianov Chuvash State University, Cheboksary, Russian Federation

## Abstract

**Introduction:**

Studying abroad often results in the change of eating and food-related practices, which may serve as manifestation of cultural distress. How much do eating behavior and daily dietary habits correlate with mental and physical health risks in South Asian students who study abroad while immersed in a different culture?

**Objectives:**

Our objective was to study the specificity of interrelations between eating behavior, salt intake, depression and its symptoms in South Asian students at universities of Uzbekistan and Russia.

**Methods:**

Using the Dutch Eating Behavior Questionnaire (DEBQ), the Salt Intake Questionnaire (Dragunov et al., 2024) and the Beck Depression Inventory (BDI-II), we surveyed anonymously 116 Indian and Pakistani students aged 18-35, who study medicine in universities of Uzbekistan (50 people) and Russia (66 people). Both groups were statistically identical in age and gender (p>.05).

**Results:**

the DEBQ showed that all of the students had indicators that met the norm. According to the Salt Intake Questionnaire, all the surveyed students revealed disordered eating patterns: 34.48% had an increased salt intake (6-10 grams/day), 65.52% showed excessive salt intake (>10 grams/day). The BDI-II revealed signs of depression of various intensity in 46.55% of the surveyed students. The correlational analysis showed that salt intake in students in Russia positively correlates with the depressive symptom of guilty feelings (r=.27; p<.05), while the general level of depression correlates with suicidal thoughts or wishes (r=.73; p<.05). We also found the connections of emotional eating with salt intake (r=.26; p<.05) and the level of depression (r=.29; p<.05); external eating behavior with salt intake (r=.29; p<.05) and the level of depression (r=.28; p<.05); as well as dietary restraint with salt intake (r=.29; p<.05). In South Asian students in Uzbekistan salt intake correlates with depressive symptoms of past failure (r=.33; p<.05), self-dislike (r=.29; p<.05) and worthlessness (r=.29; p<.05). External eating behavior in such students is interrelated both with salt intake (r=.34; p<.05), and general level of depression (r=.40; p<.05) and suicidal thoughts or wishes (r=.47; p<.05), emotional eating correlates with salt intake (r=.28; p<.05).

**Conclusions:**

Increased salt intake and certain eating styles in South Asian students in Uzbekistan and Russia have different manifestations of increased depression and suicidal ideations. Overall, due to raising blood pressure and causing cardiovascular diseases (Hunter et al., 2022) excessive salt intake interconnects somatic and mental health risks. University authorities might take into account the given pathological interconnections when planning preventive programs for international students.

**Disclosure of Interest:**

None Declared

